# Mucosal-associated invariant T cells and oral microbiome in persistent apical periodontitis

**DOI:** 10.1038/s41368-019-0049-y

**Published:** 2019-05-09

**Authors:** Haleh Davanian, Rogier Aäron Gaiser, Mikael Silfverberg, Luisa W. Hugerth, Michał J. Sobkowiak, Liyan Lu, Katie Healy, Johan K. Sandberg, Peggy Näsman, Jörgen Karlsson, Leif Jansson, Lars Engstrand, Margaret Sällberg Chen

**Affiliations:** 1 Department of Dental Medicine, Karolinska Institutet, Huddinge, Sweden; 2 Department of Microbiology, Tumor and Cell Biology and Science for Life Laboratory, Karolinska Institutet, Stockholm, Sweden; 3 Clinical Genomics Facility, Science for Life Laboratory, Solna, Sweden; 4 Department of Medicine, Karolinska Institutet, Huddinge, Sweden; 5 Clinic of Endodontics and Periodontology, Eastman Institute Stockholm, Stockholm, Sweden

**Keywords:** Microbiome, Cell signalling

## Abstract

Opportunistic bacteria in apical periodontitis (AP) may pose a risk for systemic dissemination. Mucosal-associated invariant T (MAIT) cells are innate-like T cells with a broad and potent antimicrobial activity important for gut mucosal integrity. It was recently shown that MAIT cells are present in the oral mucosal tissue, but the involvement of MAIT cells in AP is unknown. Here, comparison of surgically resected AP and gingival tissues demonstrated that AP tissues express significantly higher levels of Vα7.2-Jα33, Vα7.2-Jα20, Vα7.2-Jα12, Cα and tumour necrosis factor (TNF), interferon (IFN)-γ and interleukin (IL)-17A transcripts, resembling a MAIT cell signature. Moreover, in AP tissues the MR1-restricted MAIT cells positive for MR1–5-OP-RU tetramer staining appeared to be of similar levels as in peripheral blood but consisted mainly of CD4^+^ subset. Unlike gingival tissues, the AP microbiome was quantitatively impacted by factors like fistula and high patient age and had a prominent riboflavin-expressing bacterial feature. When merged in an integrated view, the examined immune and microbiome data in the sparse partial least squares discriminant analysis could identify bacterial relative abundances that negatively correlated with Vα7.2-Jα33, Cα, and IL-17A transcript expressions in AP, implying that MAIT cells could play a role in the local defence at the oral tissue barrier. In conclusion, we describe the presence of MAIT cells at the oral site where translocation of oral microbiota could take place. These findings have implications for understanding the immune sensing of polymicrobial-related oral diseases.

## Introduction

Apical periodontitis (AP) is a common oral inflammatory condition affecting the periodontal tissues surrounding the root-end of a tooth. A common aetiology is root canal (endodontic) infections, which trigger the host defence to induce changes in the periapical bone tissue, resulting in bone resorption. Unfortunately, AP frequently persists despite root canal treatments. Several recent epidemiological surveys report that up to 60% of adults in Western countries have more than one root-filled tooth and that signs of AP are seen among 35–50% of root-filled teeth.^1–4^ Reasons attributable to the high prevalence of AP include diffuse clinical presentations and that routine radiology screenings cover only the marginal, but not apical bone. Moreover, given that persistent AP cases are often asymptomatic, they are not always retreated.

Because of the high rate of persistent AP in the general adult population, secondary factors associated with AP, such as bacterial translocation and chronic low-grade inflammations pose a systemic health risk to patient.^5^ Spontaneous reactivation of AP as exacerbating apical abscess may also occur and spread, resulting in severe complications or mortality.^6^ Accumulating evidence further indicates associations between AP and co-morbidities, including diabetes mellitus, hypertension, and chronic liver diseases. Notably, endodontic bacteria with local inflammation were reportedly found in thrombosis of myocardial infarction patients.^7,8^ Understanding the immunological control of AP-associated microbiota is hence of importance.

Mucosal-associated invariant T (MAIT) cells belong to a group of unconventional innate-like T cells known to have broad and potent antimicrobial activity in response to metabolically active bacteria.^9^ Scattered along GI mucosal sites and the liver, MAIT cells detect microbial infection by recognising riboflavin metabolites through presentation by MR1 molecules.^10,11^ As riboflavin biosynthesis pathway is widely expressed among microbes and not in human cells, MAIT cells are therefore important gate keepers in the human immune system and are considered to play a role in diseases of microbial origin.^12–14^ Recently we have reported that MAIT cells, distributed along the buccal mucosa, are involved in the buccal mucosal immune system in healthy donors.^15^ The aim of the present study was to investigate whether MAIT cells are also a part of AP lesion defence and to explore the potential relationship between MAIT cells and AP microbiota.

## Results

### MAIT cells and cytokine signature in AP

A total of 41 patients (mean age 57 ± 17,16, 23 females and 18 male) undergoing AP surgery were included in the study cohort, of which 36 (87.8%) were non-smokers (Table 1a). Other chronic diseases were reported by 22 patients (53.7%), including endocrine (*n* = 4), heart (*n* = 18), inflammatory (*n* = 3), and neurological (*n* = 1) diseases. Regular drug intake was reported in majority of patients (Table 1b), in which cardiovascular drugs were most common followed by nonsteroidal anti-inflammatory drug (NSAID)/paracetamol, statins, and antihistamine/cortisol drugs. Moreover, those with chronic systemic diseases (100% report regular drug intake) show similar frequency of AP with progression or symptom (68%) as those without chronic systemic diseases (63%) where drug intake is less common (33%). In patients with chronic systemic diseases with AP progression and/or symptom, use of cardiovascular drugs is most common (47%) but is at same level in those without AP progression and/or symptoms. Next common drugs are NSAID/paracetamol or statins (20%–27%), with notion that use of NSAID/paracetamol is less common (0%) in patients with chronic systemic disease without AP progression/symptoms.

**Table 1A:** Patient characteristics and correlations

Variable	All patients (*n* = 41)	16S copies/µg DNA in AP tissue	
		*P*-value^a^	Correlation (Spearman *r*, *P*)
General			
Gender (female/male)	23 / 18	0.186 6	
Age (median, range)	57, 30–84		***r*** = **0.379,** ***P*** = **0.017**
Smoking (y/n)	5 / 36	0.229 7	
Existing root filling (y/n)	38 / 3		
Objective symptoms (y/n)	27 / 14	0.302 8	
Percussion (y/n)	7 / 33	0.631 7	
Palpitation (y/n)	11 / 30	0.177 2	
Fistula (y/n)	10 / 31	**0.006 5**	
Radiographic progression (y/n/no data)	12 / 12 / 17	0.116 4	
Lesion size/mm (median, range)	5.9, 2–15.4		*r* = 0.230, *P* = 0.159
Lesion growth/mm (median, range)	1, −0.9–11.1		*r* = 0.282, *P* = 0.229
Antibiotic last 3 months (y/n)	3 / 38	0.901 4	
Upper jaw/lower jaw	33 / 8	0.798 1	

y/n*,* yes/no

^a^Mann–Whitney test

Statistically significant comparisons (*p* < 0, 05) are displayed in bold font

**Table 1B:** Overview of chronic systemic conditions and drug intake among participants

Patient parameter	All (*n* = 41)	Chronic systemic disease (*n* = 22)		No chronic systemic disease (*n* = 19)	
Sign of AP progression symptom		No (32%)	Yes (68%)	No (37%)	Yes (63%)
Drug intake	71%	100%	100%	43%	33%
ACE-inhibitors, beta or calcium channel blockers, angiotensin II antagonist	27%	43%	47%	14%	0
NSAID, paracetamol	15%	0	20%	14%	17%
Statins	17%	29%	27%	0	8%
Antihistamines, cortisol	10%	29%	0	14%	8%
Others	2%	0	7%	0	0

ACE, angiotensin-converting-enzyme; NSAID, nonsteroidal anti-inflammatory drug

**Fig. 1:**
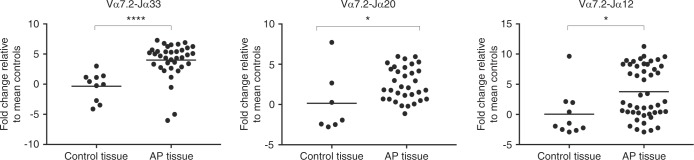
Tissue expression of Vα7.2-Jα33, Vα7.2-Jα20, and Vα7.2-Jα12 in AP lesions and gingival control biopsies. The expression of Vα7.2-Jα33, Vα7.2-Jα20, and Vα7.2-Jα12 was significantly increased in AP tissues compared to donor-matched gingival control tissues. Expression levels were measured by reverse transcription**-**qPCR and graphed as the log2 fold-change relative to GAPDH expression. Statistical analysis was performed using Student’s *t*-tests. ns indicates non-significant, *, **, ***, and **** indicates *P* < 0.05, < 0.01, < 0.001, or < 0.000 1, respectively

**Fig. 2:**
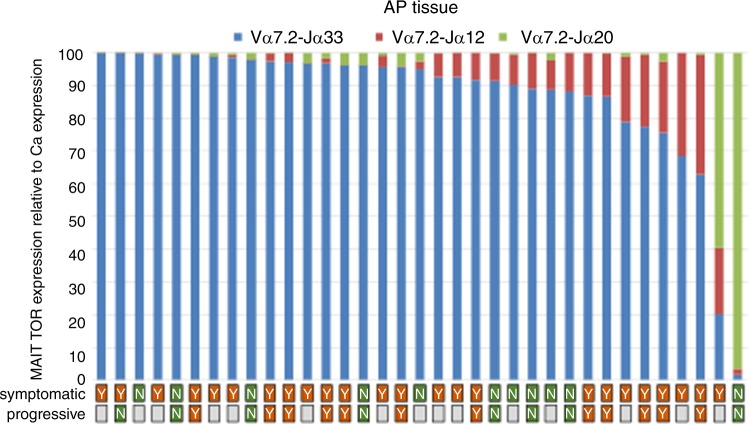
The MAIT distribution. TCR typing of AP MAIT cells confirmed the Vα7.2-Jα33 as the dominant TCR rearrangement in the AP tissue

To examine if MAIT cells are present in AP, surgically removed AP were screened for semi-invariant T-cell receptors (TCRs)—Vα7.2 and joining genes Jα33, Jα12, and Jα20 known to be MAIT cell markers. Gingival tissues (*n* = 10) collected from peri-surgical sulcus area were used as non-AP donor/patient-matched oral tissue controls. MAIT cell semi-invariant TCRs Vα7.2-Jα33, Vα7.2-Jα20, and Vα7.2-Jα12 were significantly expressed in AP tissues compared to control tissues (Fig. 1). Among which the Vα7.2-Jα33 recombination was found to be the most abundant (*P* = 0.000 1), followed by Vα7.2-Jα12 (*P* = 0.014), and Vα7.2-Jα20 (*P* = 0.022) as shown in the distribution of the accumulated TCR expression (Fig. 2). The distribution of these three TCR types in AP did not seem to correlate with signs of growth/progression or clinical symptoms (Supplementary Fig. Ia).

**Fig. 3:**
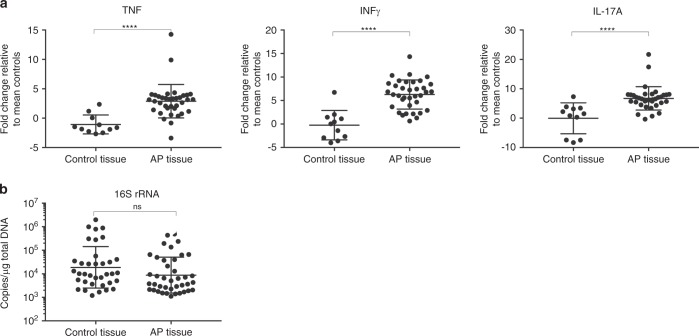
**a** The expression of TNF, IFN-γ, and IL-17A in AP lesions and gingival control biopsies. The expression of TNF, IFN-γ, and IL-17A was significantly increased in AP tissues compared to gingival control tissues harvested from same patients. **b** Quantification of tissue-associated microbial 16S DNA. Tissue 16S rRNA gene copy number per milligram total genomic DNA isolated from indicated tissue sample. Statistical significance of comparisons between groups was made using Mann–Whitney test

Activated MAIT cells are known to secrete tumour necrosis factor (TNF), interferon (IFN)-γ, and interleukin (IL)-17A,^16,17^ hence the tissue expression levels of these cytokines was measured. Shown in Fig. 3a, the AP tissues had significant expression of TNF (*P* < 0.000 1), IFN-γ (*P* < 0.000 1), and IL-17A (*P* < 0.000 1) compared to control gingival tissues. Interestingly, the TNF expression was significantly higher in AP cases that showed disease progression (*P* = 0.025 5) (Supplementary Fig. Ib).

### Bacterial 16S rRNA gene levels in AP lesions

Next, the AP tissues were screened for the bacterial load by qPCR quantification of the 16S rRNA gene. The presence of bacteria in AP tissue was found to be at comparable levels to the gingival control tissues (Fig. 3b). Notably, the median 16S copy number was >10-fold higher in AP tissues with fistulas (*P* = 0.006 5) compared to AP tissues without fistulas (Table 1). Symptoms, disease or other clinical parameters had no significant impact on 16S gene copy numbers (Table 1). Moreover, a weak but significant correlation (Spearman *r* = 0.379, *P* = 0.017) was found between patient age and the absolute 16S rRNA gene copy number per µg gDNA in AP tissue, but not in the control gingival tissues (Table 1).

### Flow cytometry analysis of tissue MAIT cells

**Fig. 4:**
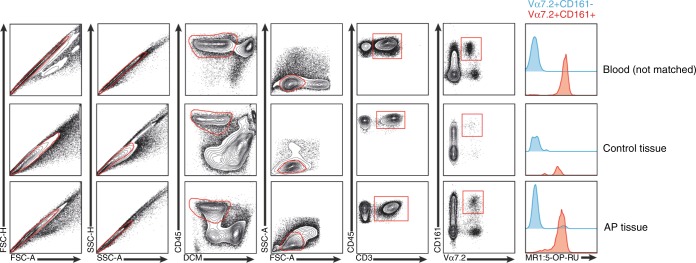
FACS analysis of single suspension cell isolates from AP and gingival control tissues. Gating strategy to define MAIT cells (Va7.2 + CD161^hig^^h^ ) within the CD45 + subset in blood, control tissue, and AP granuloma and representative histogram of MR1:5-OP-RU tetramer stain of Vα7.2 + CD161^high^ (MAIT) and Va7.2 ^+^ CD161^low^^-^ cells (*n* = 2)

**Fig. 5:**
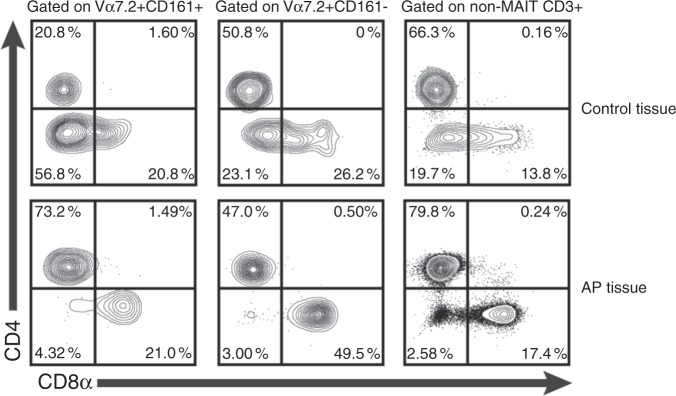
Contour plot of CD8α vs. CD4 expression in MAIT (Vα7.2 + CD161^high^) and non-MAIT CD3^+^ cells

In addition to cell-surface expression of the Vα7.2 receptor, human MAIT cells express high levels of CD161.^18^ In a secondary set of AP tissue (*n* = 6), single cell suspensions were prepared after collagen digestion, stained, and analysed by multicolour flow cytometry. Staining was performed with an antibody panel, including the 5-OP-RU MR1 tetramer to ensure that Vα7.2^+^161^high^ cells defined by the gating strategy (Fig. 4) were bona fide MAIT cells. As reference staining gingiva tissue and peripheral blood (*n* = 2) were used. Our data indicates that in AP tissue, Vα7.2^+^CD161^high^ MAIT cells appear at similar levels as in peripheral blood and that unlike gingiva, AP-associated MAIT cells consist mainly of CD4^+^ subset with markedly lower levels of CD4^-^/CD8^-^ double-negative MAIT cells (Fig. 5). Intracellular staining with IL-17A or IFN-γ was seen in a proportion of MAIT cells in AP but no statistics could be provided due to the limited number of lymphocytes that could be acquired from gingival tissues for this assay (data not shown).

### High interpersonal variation in AP tissue microbiome

**Fig. 6:**
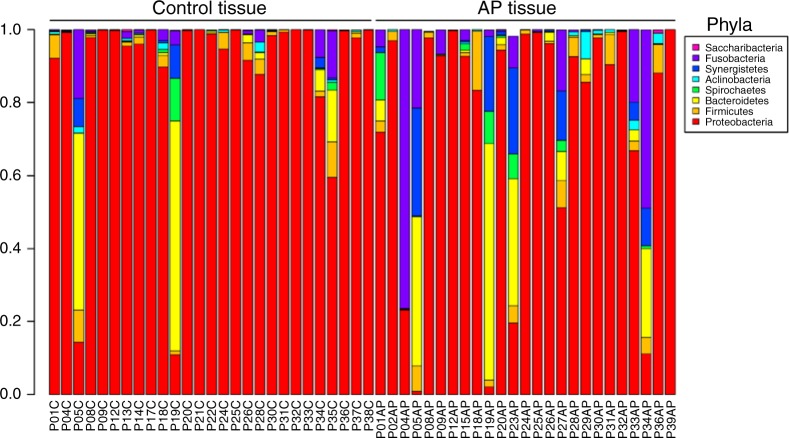
Taxonomic profiles at the phylum levels. Bar plots displaying relative abundance of OTUs within an individual sample of gingival control tissues and AP tissue biopsies at the phylum level

**Fig. 7:**
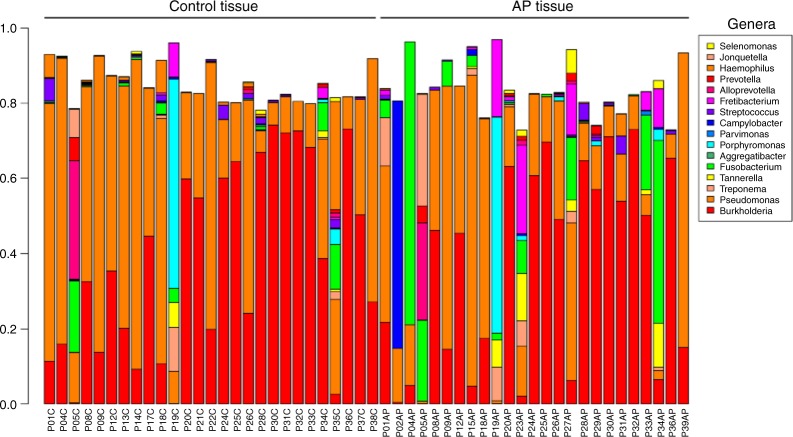
Taxonomic profiles at the genus levels. Bar plots displaying relative abundance of OTUs within an individual sample of gingival control tissues and AP tissue biopsies at the genus level

**Fig. 8:**
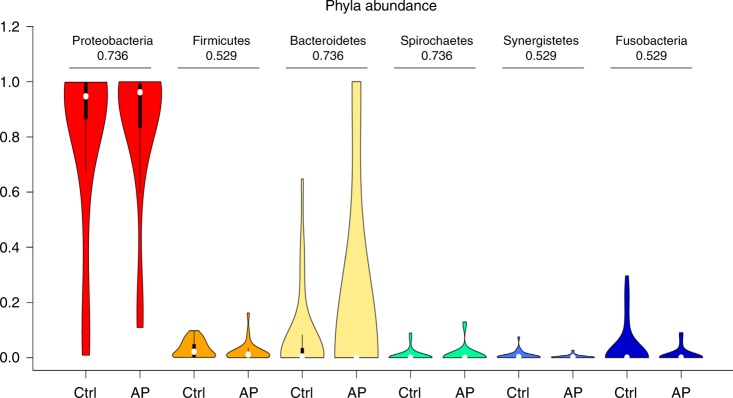
Taxonomic abundances at the phylum levels. Violin plots showing the comparison of the relative abundance of selected bacterial taxa at phylum level between gingival control tissue samples and AP tissue samples with adjusted *p*-values being shown

**Fig. 9:**
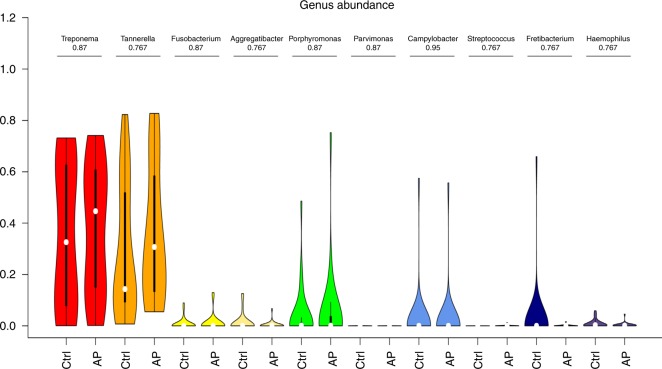
Taxonomic abundances at the genus levels. Violin plots showing the comparison of the relative abundance of selected bacterial taxa at genus level between gingival control tissue samples and AP tissue samples with adjusted *p*-values being shown

We next examined the microbial composition in the collected tissue samples by 16S rRNA gene-based microbiome sequencing. Following library production and bioinformatic processing, a total of 52 (25 AP + 27 gingival control tissues) tissue samples qualified for further downstream comparisons. The obtained reads were clustered at 99% sequence identity into 642 operational taxonomic units (OTUs), which were assigned to taxa to obtain bacterial profiles. Among these compositional profiles, we observed a large interpersonal variation in the diversity and abundance of bacterial taxa in AP lesions, as well as in gingival tissues with predominating taxa belonging to the phyla *Proteobacteria, Bacterioidetes, Firmicutes, Fusobacteria, Spirochaetes*, and *Synergistetes* (Fig. 6). At the genus level, the predominating OTUs were assigned to *Burkholderia-Paraburkholderia, Pseudomonas, Achromobacter*, and *Fusobacterium* (Fig. 7). No statistically significant differences were found between the relative abundance of bacterial taxa at phylum (Fig. 8) or genus (Fig. 9) level when comparing gingival control tissue and AP tissue. Linear discriminant analysis (LDA) effect size (LEfSe)^19^ was used to better explore the differences in bacterial profiles that characterise control gingival tissue and AP tissue, but no statistically significant differentially abundant bacterial taxa were found (data not shown). A comparison on alpha diversity, e.g., the richness, in these samples showed no statistical significance between the groups (data not shown), but a significant difference was found at the beta-diversity level showing significant subgroup distances dependent on symptom and progression of lesion. Shown in Table 2 and Supplementary Fig. II, it was found that unlike gingival control biopsies, the progressive and symptomatic AP microbiomes show significantly higher beta-diversity (*P* = 0.008 75 and 0.000 85, respectively) compared to total between-sample diversity (All). On the other, samples within same patients in general show significantly lower beta-diversity (*P* = 0.000 26).

**Table 2:** Beta-diversity analysis

Sample type	Patients	Mean distance	Raw *P*-value	Adjusted *P*-value
All	Between patients	0.547	NA	NA
**All**	**Within patients**	**0.250**	**0.000 03**	**0.000 26**
Control tissue	Progressive	0.534	0.903 20	0.903 20
Control tissue	Non-progressive	0.534	0.866 69	0.903 20
Control tissue	Symptomatic	0.484	0.036 34	0.081 77
Control tissue	Asymptomatic	0.471	0.071 66	0.128 98
**AP tissue**	**Progressive**	**0.673**	**0.002 92**	**0.008 75**
AP tissue	Non-progressive	0.626	0.346 95	0.520 43
**AP tissue**	**Symptomatic**	**0.643**	**0.000 19**	**0.000 85**
AP tissue	Asymptomatic	0.507	0.512 65	0.659 12

Significant differences are highlighted in bold

### Integrated analysis of MAIT cell signature and AP microbiome

**Fig. 10:**
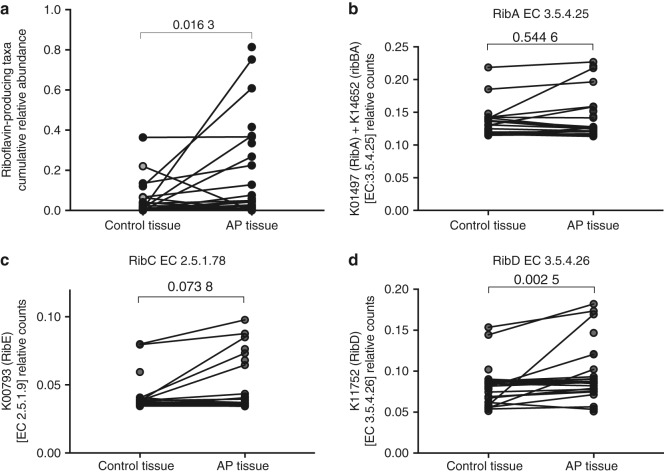
Identification of the microbiota with known riboflavin-producing activity among annotated OTUs. **a** Cumulative relative abundance of riboflavin-producing bacterial taxa (predicted from literature and KEGG database search) in oral control tissue and paired AP tissue. Relative gene counts of **b** RibA, **c** RibE, and **d** RibD as inferred from 16S sequence data by PICRUSt in control tissue compared to AP tissue. Wilcoxon matched-pairs signed rank test (two-tailed) was used to assess statistically significant difference between the two groups, *p*-values are displayed

To further elucidate the relationship between bacteria found in APs and the tissue-associated MAIT cells, a systematic search was performed in the KEGG database and literature to identify bacterial taxa with known endogenous riboflavin biosynthetic pathway (RBP), to cross-check against the annotated OTUs from our sequencing result. In the AP and gingival tissue samples, a total of ten bacterial families/genera known to harbour genes that encode the paralogs of the RBP enzymes required for a functional riboflavin biosynthesis pathway were identified (Table 3).^20^ As noted, despite that these riboflavin-producing taxa were similarly prevalent both in AP and gingival tissues, the cumulative relative abundance of these taxa was significantly higher in AP tissues as compared to their paired gingival tissues (*P* = 0.016 3) (Fig. 10a). Similarly, inferred functional metagenomics using PiCrust^21^ showed statistically significant enrichment of riboflavin biosynthesis pathway genes RibC (*P* = 0.073 8) and RibD (*P* = 0.002 5), but not RibA (*P* = 0.544 6) in the AP microbiome compared to gingiva (Fig. 10b–d).

**Table 3:** Bacteria with known functional riboflavin biosynthesis pathways

Phylum	Family or genus	Prevalence in AP lesions/%	Prevalence in control tissues/%
Actinobacteria	Corynebacterium	42.9	50.0
Bacteriodetes	Bacteroides	3.6	0.0
	Prevotella	57.1	67.9
	Rikenellaceae	25.0	10.7
Firmicutes	Streptococcus	57.1	78.6
	Anaerococcus	10.7	14.3
	Erysipelotrichaceae	3.6	3.6
Fusobacteria	Fusobacterium	71.4	75.0
Proteobacteria	Ralstonia	14.3	17.9
	Bilophila	3.6	0.0
	Desulfovibrio	7.1	0.0
	Campylobacter	39.3	42.9
	Enterobacteriaceae	85.7	82.1
	Acinetobacter	3.6	3.6
Synergistetes	Fretibacterium	42.9	39.3

All AP lesions and control tissue contained multiple bacterial taxa that are predicted to have functional riboflavin biosynthesis pathways, specifically containing a 6,7-dimethyl-8-ribityllumazine (RL-6,7-diMe) synthase gene (EC 2.5.1.78) required for the production of the main MR1-binding ligand RL-6,7-diMe^11,52^

**Fig. 11:**
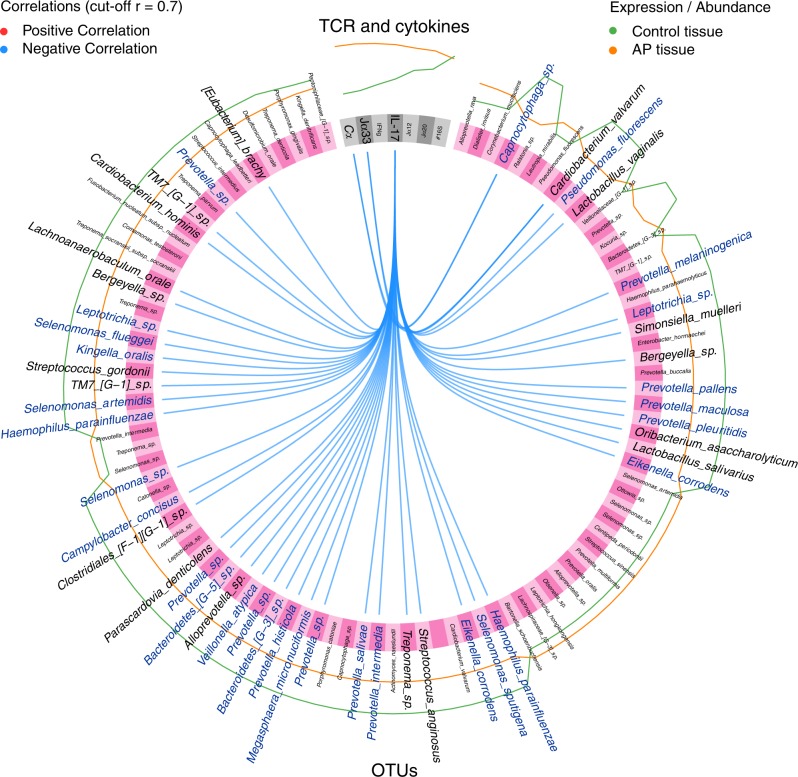
Circos plot showing correlation analysis of immunological parameters with microbiota data. Sparse partial least squares discriminant analysis (sPLS-DA) was used to identify a first and second component based on TCR- and cytokine expression levels or absolute 16S rRNA counts and OTU relative abundance. The most discriminative features that were selected by the model from TCR- and cytokine expression data (grey) and OTU abundance (pink) are shown, where the outermost lines represent the feature abundance or expression level in samples from control tissue (green) and AP tissue (orange). At a correlation cutoff of 0.7, only negative correlations (blue lines) between the features were found. Bacterial taxa presumed to have functional riboflavin biosynthesis pathways are highlighted in blue font

Next, we examined the relationships between T-cell- and microbial factors, using a sparse partial least squares discriminant analysis (sPLS-DA),^22^ which creates linear combinations (so-called components) from features selected by the model that best discriminate differences between two classes, e.g., AP or gingival tissue. Features such as OTU abundance and T-cell/cytokine expression levels were selected by this model, and the inter-feature correlations at a correlation cutoff of 0.7 are illustrated in a Circos plot showing the first and second component (Fig. 11). Consistent with the observed MAIT cell and cytokine signature in AP, the analysis indicated that differences between AP and gingiva were explained by multiple negative correlations between various bacteria OTUs and the IL-17A, Cα and Vα7.2-Jα33 expression. Notably, among those negatively regulated—*Capnocytophaga, Cardiobacterium, Pseudomonas, Lactobacillus, Prevotella*, *Leptotrichia, Simonsiella, Bergeyella, Oribacterium*, *Selenomonas*, *Eikennella*, *Streptococcus, Treponema, Megasphaera, Veilonella, Alloprevotella, Parascardovia, Clostridiales, Campylobacter*, *Haemophilus, Kingella, Leptotrichia*, and *Eubacterium* (Fig. 11, labelled blue), the majority also appear to encode functional riboflavin biosynthesis pathways as indicated by the KEGG database.

## Discussion

In the present study, we investigated AP lesions with a focus on tissue inflammation and microbiome composition. In line with previous reports that AP tissues contain conventional T cells, including Tregs,^23,24^ our findings show for the first time that AP lesions also are infiltrated by MAIT cells characterised by unique TCRs composed of Vα7.2-Jα33/20/12 α-chain rearrangements. Moreover, AP tissues show a partial resemblance to that reported in blood MAIT cells, i.e., mainly of Vα7.2-Ja33 rearrangement. However, phenotypically the AP-associated T cells appear to consist mainly of CD4^+^ subset, whereas MAIT cells in blood or oral mucosal tissues mostly are CD8αα^+^ and CD4^-^/CD8^-^ double-negative, respectively.^15^ Among the concomitant cytokines expressed, our data further suggest that TNF was associated with progression of AP and that IL-17A correlated inversely with multiple bacterial taxa within AP microbiome that likely utilise the riboflavin pathway. This supports earlier reports that functional IL-17RA signalling protects against infection-induced AP and bone loss,^25^ and that TNF and IL-17R signalling are important in bone destruction in periodontitis.^26^ Of note, T cells that can produce IL-17A include Th17 cells are known to play an important role in protective immunity through immune surveillance and maintenance of (mucosal) barrier integrity.^27,28^ Although Th17 cells have a classical T-cell receptor repertoire that is not limited to the Vα7.2-Ja33 rearrangement like MAIT cells, we cannot rule out any potential impact they may have on the AP microbiome, and this area thus deserves to be further investigated in future studies.

MAIT cells respond to antigenic stimulus with an innate-like speed and produce proinflammatory cytokines TNF, IFN-γ, and IL-17.^18,29–31^ While MAIT cells are not the only cells that can produce these cytokines, we have recently shown the oral mucosal MAIT cells in fact produce them upon bacteria re-stimulation in functional assays using explanted oral mucosa from healthy young individuals.^15^ Therefore, it is a reasonable interpretation that MAIT cells being present in AP might contribute to the observed cytokine expression. Additionally, MAIT cells can eliminate cells that are infected by riboflavin-pathway expressing microbes and inhibit their intracellular microbial growth.^32^ AP tissue showed significant MAIT cell and inflammatory cytokine transcript signatures compared to gingival tissue controls, and exhibited a significant amount of oral bacteria known to express riboflavin biosynthesis pathways. We reported recently that the percentage of MAIT cells in the oral mucosa ranged between 0.1 and 7% of total T cells in peripheral blood.^15^ Here, flow cytometric analysis of AP-associated MAIT cells demonstrated that they occur with similar frequency. Our results also indicate a significant but strong negative correlation between bacterial taxa, especially riboflavin-pathway expression taxa, and IL-17 and Vα7.2-Jα33 expression, suggesting the host MAIT cell defence mechanism is upregulated. Despite this MAIT cell activation, the bacterial load in AP tissues is not sufficiently reduced hence allowing persistence of AP and disease progression. A notable increase of conventional T cells (Cα and CD3^+^) seen in AP also suggests that other cell types, such as previously reported regulatory T cells, could further help to dampen the inflammation.^23^

Persistent AP in root-filled or obliterated roots is commonly related to growth of unremoved bacteria left from the primary AP.^33^ As MAIT cells are known to sense bacterial activity through recognition of riboflavin metabolites, the composition and metabolic capacity of the microbiota in these AP tissues was of interest. As control tissues, we deliberately chose oral tissues from the surgically detached gingival flap of same patients, as gingival tissues have similar physiological environment as AP, i.e., in an oral environment constantly exposed to patient´s own oral microbiota. Interestingly, our quantitative analysis indicates that AP and gingival tissues had similar bacterial 16S gene copy numbers, but clinical factors such as fistula and high patient age associated with increased 16S bacterial load in AP tissues but not in control tissues, indicating that AP microbiome is under influence of these factors. Although a weak correlation was seen with high patient age, microbial translocation in elderly patients is of further health risk.

Our qualitative analysis further showed that the tissue microbiota in AP and gingival tissue were highly personal and were of high microbial diversity consisting of multiple taxa assigned as oral microbiome. This is in line with previously reported observations^34^ that the microbiota commonly occurring inside the root canals of AP-associated teeth has a polymicrobial composition. This is strengthened by the increased intra-sample diversity in progressive and symptomatic AP, which suggests that a healthy microbiome is associated to less severe AP.

To eliminate potential artifacts from the sequencing data, reads from negative PCR amplification controls were used. This ensured that the majority of those bacterial reads left in our samples do not come from the reagents. The best available control samples from the surgical field hypothetically were the gingival flaps, which were treated in exactly the same way. However, it is hard to expect them having an entirely different microbiome from the AP lesions, as biologically this would imply in a strict separation between the microbiomes in the AP and the surrounding oral tissue, which is rather unlikely. It is also not possible to rule out potential micro-communications between marginal- and apical periodontal space, but to further interrogate this is beyond the scope of this study. Given that human oral cavity is constantly exposed environmental challenges, water from dental unit, dental grade materials, and even sterile saline of surgical grade etc. are also likely to contribute with bacteria that may persist on oral sites following dental treatments with environmental bacteria that may persist on oral sites following dental treatments.

Here, taxa found in AP included Fusobacterium nucleatum, Treponema denticola, Tannerella forsythia, Porphyromonas gingivalis, as well as Tanerella sp, Enterococcus sp., Prevotella sp., Parvomonas, Campylobacter, Streptococcus sp, Fretibacterium and Haemophilus. Enterococcus faecalis, which is often associated with treatment-resistant AP, was found in only one of our cases reinforcing a recent report by Siqueira et al., that E. faecalis is sparsely found in AP tissues or the apical root canal system.^35,36^ In addition to these taxa, both AP and gingival tissues also contained substantial amounts of *Pseudomonas fluorescens* and *Burkholderia cepacia*, which are generally considered environmental bacteria and nonpathogenic bacteria for humans. In the oral cavity, they have been found to be associated with dentures,^37^ persistent periradicular lesions, as well as chronic maxillary sinusitis.^38^ The latter is of interest, as the majority of lesions examined here were from upper jaw and could potentially serve as a source to sinusitis of odontogenic origin.^6^ *P. fluorescens* was abundant in the saliva of nearly 50% of transplant subjects while being nearly absent from nontransplant controls.^39^ *P. fluorescens* has also repeatedly been cultured from respiratory and gut wall specimens and hence may have possible roles in pathogenesis of respiratory and gut diseases.^40^ Furthermore, our current finding is supported by recent metaproteome analysis that components from these bacteria are found in both root apex and periapical lesion of AP requiring surgical treatment.^41^ In our present study, the riboflavin-producing function was further considered, and we could conclude that AP tissue microbiota were found to contain a significantly increased proportion of riboflavin-producing taxa, hence possibly supporting MAIT cell infiltration in persistent AP.

The present study provides for the first time a compiled immunological and microbiome profile of AP. The clinical profiles of cases were documented with a standardised questionnaire for participating clinicians and patients to allow consistent definition of symptoms and disease progression, which is an additional strength of this study. This study is not without limitations, for instance the tissue sample size allowed measurements relying mostly on molecular methods, and the 16S gene-based microbiota analysis does not allow distinction between live and dead bacteria, nor is it possible to exclude involvement of other microbes like fungi. Chronic systemic diseases and regular drug intake in this present study cohort may confound our findings; radiographic progression data not available for all patients due to national radiation safety authority´s regulations, hence clinical signs of symptom were included as additional parameter of interest to support the data interpretations. Although vast majority of our cases were root-filled previously, the heterogeneity of clinical manifestations could also have impacted on the structure and composition of microbiota that contributed to the interpersonal AP microbiome variation observed. Study cohorts with better homogeneity will be required in future studies using stricter inclusion/exclusion criteria.

Unlike earlier studies, the current study employed an integrated view of immunological and microbiome data to illuminate interactions between immune parameters and tissue microbial composition in AP, and identified for the first time a potential role of MAIT cells in a disease that manifests in oral cavity. Given that MAIT cells are present but not enriched in AP tissues despite the presence of ectopic bacteria, regulatory mechanisms provided either by the host or pathogen encoded factors could prevail to supporting the persistent tissue inflammation, hence further motivates therapeutic interventions of persistent AP.

## Materials And methods

### Ethics statement

The study was performed in accordance with the Declaration of Helsinki and the current legislation in Sweden following approval from the Karolinska Institutet Ethical Research Board. Collection of blood and biopsies was approved by the Regional Ethics Board in Stockholm and written informed consent was obtained from each patient.

### Subjects

Patients with persistent AP admitted to the Endodontics Specialist Clinic of Karolinska Institutet or Eastman Institute to undergo endodontic surgery during 2014–2017 were consecutively enroled into this cross-sectional study. Indications for inclusion were radiological findings of AP and/or symptoms associated with an obstructed canal, radiologically verified persistent AP lesion (>12 months) without ongoing exacerbation.

The vast majority of included AP cases were root-filled previously and almost half of AP lesions showed symptoms or signs of growth in clinical radiographic evaluation. A standardised examination protocol was used to define the cases, where specific parameters included lesion size, presence of fistula, root-filling, or marginal periodontitis, and presence (symptomatic) or absence of (asymptomatic) of provocable pain response to clinical percussion or palpation. Medical data collected include systemic disease diagnoses and usage of medications.

AP lesions were obtained by curettage during standard mucoperiosteal flap surgery. Alcohol/ethanol (70%) was used in pre-operation disinfection of the skin and lips with closed mouth, sterilised drapes were then applied to isolate the disinfected area with sealed adhesion. Three-corner flap was designed around the surgical site and drills were used to expose the lesion with sterile saline (B. Braun Melsungen, Melsungen, Germany) to avoid bone damage under microscope. The AP lesion was removed by sterile curettes and transferred immediately to pre-prepared RNAlater solution (Applied Biosystems, Foster city, CA, USA) in a sterilised container under assistant supervision. As a control, oral tissues from the detached gingival tissue flap (3 mm × 3 mm) were removed with Biopsy Punch (Kai industries, Oyana, Gifu, Japan) and transferred immediately as above. Great care was taken to avoid saliva contamination of the surgical site during the entire sample collection. After this, root resection as well as the root-end filling were done to reduce the recurrence of the disease before suturing.

### MAIT TCR and cytokine expression quantification using reverse transcription quantitative PCR

AP tissues and gingival control tissues were placed in RNAlater solution (Applied Biosystems, Foster city, CA, USA) immediately following tissue biopsy, stored overnight at 4 °C, and thereafter transferred to −80 °C for (Applied Biosystems, Foster city, CA, USA) subsequent DNA and RNA isolation. Homogenisation of AP and control tissues was performed using steel-bead matrix tubes and a table-top Fast-Prep homogeniser by two sequential centrifugations for 20 s at speed 6.5 units (Qbiogene, Irvie, CA, USA). Afterwards, the lysate was centrifuged for 3 min at maximum speed and the supernatant was transferred to an Allprep DNA spin column (Qiagen, Valencia, CA, USA) and stored at 4 °C for DNA purification. In parallel, RNA was extracted and purified on RNeasy Spin Columns (Qiagen, Valencia, CA, USA), and treated with DNAse H, eluted in RNase-free water and quantified using Nanodrop. The iScript cDNA synthesis kit (Bio-Rad Laboratories) was used according to manufacturer’s instructions to reverse transcribe 250 ng total RNA per sample. Reverse transcription quantitative PCR (qPCR) was performed (5 min at 95 °C, then 40 cycles of 10 s at 94 °C, 30 s at 58 °C or 60 °C, and 27 s at 72 °C) on a 7500 Fast Real-Time PCR system (Applied Biosystems) with 1 μL cDNA, 200–500 nmol· L^−1^ forward and reverse primer, 5 μL SsoAdvanced™ Universal SYBR® Green Supermix reagent (Bio-Rad Laboratories) and nuclease-free water up to a total volume of 10 μL per reaction. The expression of Vα7.2-Jα33, Vα7.2-Jα20, Vα7.2-Jα12, TCR Cα, TNF, IFN-γ, IL-17A, as well as house-keeping gene glyceraldehyde-3-phosphate dehydrogenase (GAPDH) transcripts were quantified using respective primers with indicated annealing temperature: Vα7.2-Jα33 5′-GTCGGTCTAAAGGGTACA-3′ and 5′-CCAGATTAACTGATAGTTGCTA-3’(58 °C); Vα7.2-Jα20 5′-AGTCGGTCTAAAGGGTACAGTT-3′ and 5′- CAGTTACTGTGGTTCCGGCT-3′ (60 °C); Vα7.2-Jα12 5′-AGTCGGTCTAAAGGGTACAGTT-3′ and 5′-GGTCCCACTCCCGAAGAT-3′ (58 °C); TCR Cα 5′-ACGCCTTCAACAACAGCATTA-3′ and 5′- TCAGGAGGAGGATTCGGAAC-3′ (58 °C); TNF 5′- GACAAGCCTGTAGCCCATGT-3′ and 5′-TCTCAGCTCCACGCCATT-3′ (58 °C); IFN-γ 5′-TCGGTAACTGACTTGAATGTCCA-3′ and 5′-TCGCTTCCCTGTTTTAGCTGC-3′ (58 °C); IL-17A 5′-AGATTACTACAACCGATCCACCT-3′ and 5′-GGGGACAGAGTTCATGTGGTA-3′ (58 °C). For each run, melting curves and melting temperatures were examined to validate specificity of the intended amplicon. Raw qPCR data were analysed using the 7500 Software v2.3 (Applied Biosystems) and gene transcript expressions were calculated relative to GAPDH gene expression using the formula: 2^ (*C*_T_ of reference − *C*_T_ of gene). Comparison analysis was performed using unpaired two-tailed *t*-tests, and statistical significance was defined as *P* < 0.05.

### Bacterial 16S rRNA gene quantification

Standard curves for the bacterial 16S rRNA gene were generated using tenfold serial dilutions from purified genomic DNA obtained from *E. coli* ATCC 25922 to allow 16S copy number quantification. Quantitative PCR conditions were as follows: 95 °C for 10 min, and 40 cycles of 95 °C for 15 s and 60 °C for 45 s on a 7500 Fast Real-Time PCR system (Applied Biosystems) with each reaction consisting of 5 ng total DNA template, 500 nmol·L^−1^ forward primer (5′-TGGAGCATGTGGTTTAATTCGA-3′) and 500 nmol·L^−1^ reverse primer (5′-TGCGGGACTTAACCCAACA-3′), 200 nmol·L^−1^ TaqMan probe (5′-FAM-CACGAGCTGACGACARCCATGCA-TAMRA−3′).^42^ Ten microlitres Fast Universal PCR Master Mix (Applied Biosystems) and nuclease-free water up to a total volume of 20 μL per reaction was used. Each reaction was run with technical replicates. Raw qPCR data were analysed using the 7500 Software v2.3 (Applied Biosystems) and 16S rRNA copies per ng total DNA was calculated using the mean *C*_T_ values per sample.

### Flow cytometry analysis

An additional set of AP and control gingival tissues (*n* = 6) were collected and stored in sampling medium (serum-free RPMI-1640 with 5 mmol·L^−1^ HEPES (GE Life Science, Sunnyvale, CA, USA), supplemented with 0.5 μg·mL^−1^ Normocin (Invivogen) and 50 μg·mL^−1^ gentamicin (Life Technologies, Carlsbad, CA, USA). Samples were transferred to fresh sampling medium containing DNase I and Collagenase A (Roche, Basel, Switzerland), both at 1 mg mL^−1^. Samples were then incubated for 1 h at 37 °C with shaking (800 r·min^−1^). After incubation, FBS (Sigma-Aldrich, St. Louise, Missouri, USA) was added to a final concentration of 10% to stop the reaction. Remaining solid tissue was pressed through a 100 μm cell strainer and the resulting cells, together with cell suspension, were transferred to a fresh tube. Cells were washed in PBS and incubated in red blood cell lysis buffer for 10 min at room temperature (RT). Finally, the cells were washed in PBS once more, counted and stained. Cells in suspension were stained for surface antigens in FACS buffer (PBS + 2 mmol·L^−1^ EDTA + 2% FCS), and thereafter washed and permeabilized using the transcription factor fixation and permeabilization buffer (BD biosciences, Franklin lakes, NJ, USA) for 30 min at 4 °C. The cells were washed twice in transcription factor wash and permeabilization buffer (BD biosciences, Franklin lakes, NJ, USA), then stained for intracellular antigens in the same buffer for 20 min at 4 °C. After staining, cells were washed and analysed using a BD LSRFortessa flow cytometer. All antibodies were purchased from BioLegend, BD Pharmingen, Beckman Coulter, BD Horizon, or eBioScience. CD45 clone HI30 (Alexa fluor 700), TCR-Vα7.2 clone 3C10 (PE), CD161 clone DX12 (PE-Cy5), CD3 clone OKT3 (Brilliant violet 650), CD69 clone TP1.55.3 (ECD), CD8α Brilliant violet 570, CD103 clone BerACT8 (Brilliant ultraviolet 395), CD4 clone OKT4 (Brilliant violet 71), TNF (clone Mab11) PE-Cy7, IL-22 clone IL22JOP (APC), IL-17A clone BL168 (Brilliant violet 421), and IFN-γ clone 4S.B3 (Brilliant violet 785) were used in this study. In addition to antibodies, cells were stained with LIVE/DEAD Aqua Fixable Dead Cell Stain (Thermo Fisher Scientific, Waltham, Massachusetts, USA).

### Bacterial 16S rRNA library preparation and sequencing

Sequencing libraries were prepared from each of the DNA extractions using up to 200 ng or 20 µL of DNA template per 50 µL reaction using a modified version of the Illumina amplicon library protocol. In total, 25 PCR cycles were carried out using V3-V4 specific primers (341F, 5′-CCTACGGGNGGCWGCAG-3′ and 805R, 5′-GACTACHVGGGTATCTAATCC-3′).^43^ Thereafter, 5 µL of cleaned amplicon from the first PCR were used as template for the second PCR, where Illumina sequencing handles are added to the amplicons. The reaction was run for 13 cycles and cleaned again before being pooled at equimolar amounts. All finished libraries were sequenced on a MiSeq instrument (Illumina Inc, Carlsbad, CA, USA). Accordingly, 69 of the prepared libraries produced a library, here defined as > 2 000 reads. For these, the average number of reads was 30 335.

### Bioinformatics processing of reads

Bases below a phred score of 25 were removed from the 3′-end of the reads using Usearch v.7.^44^ Forward reads shorter than 280 bp and reverse reads shorter than 230 bp were removed. The remaining reads were merged with Usearch and reads with >4 expected errors along the full length of the read were discarded. Reads were then dereplicated and unique reads discarded at this step. Given that environmental bacteria DNA may give rise to artifacts. Therefore, to eliminate potential artifacts, PCR amplification negative controls were sequenced and reads in the samples with >99.5% identity to the 5 most common OTU in the negative PCR control were removed. The remaining reads were mapped to the Greengenes tree^45^ with 99% identity and reads that didnot match to the reference were discarded. OTU were quantified by mapping the merged reads back to the reference reads with Usearch v.7^46^ at 99% identity. After this process, 52 samples having >2 000 reads remained, with an average of 5813 reads in each. For each OTU, the 16S centroid sequence was aligned against the Human Oral Microbiome Database (HOMDB)^47^ using RefSeq Version 14.5 to confirm the annotations. The Greengenes tree was used to build a simulated metagenome using PiCrust v.1.1.0.^21^ For a more detailed classification of the reads than allowed by Greengenes, SINA v.1.2.13^48^ was used with the NR99 v. 128 Silva database,^49^ with a minimal identity cutoff of 95% and LCA quorum of 80%.

### Biostatistics

All statistical analyses were performed in R v. 3.3.1 (R Core Team, 2016) unless otherwise stated. Data analyses and figures generation were done with packages Vegan 2.4–1,^50^ Vioplot 0.2, Pheatmap 1.0.8 (Kolde, 2015) and mixOmics 6.3.1.^51^ All pairwise comparisons were based on Wilcoxon’s test. Diversity measures used were Shannon’s entropy for alpha diversity and Bray–Curtis dissimilarity for beta-diversity. Differentially abundant OTU or KEGG categories were searched for using LEfSe^19^ and MaAsLin on a galaxy environment.

## Supplementary information

Supplementary Figure I

Supplementary Figure II

Supplementary Figure legend
