# Supplementary figures and images for: Mucosal-associated invariant T cells and oral microbiome in persistent apical periodontitis
Source: Int J Oral Sci. 2019 May 9;11(2):16. doi: 10.1038/s41368-019-0049-y (PMC6506549; doi:10.1038/s41368-019-0049-y)

Supplementary Figure. I

a

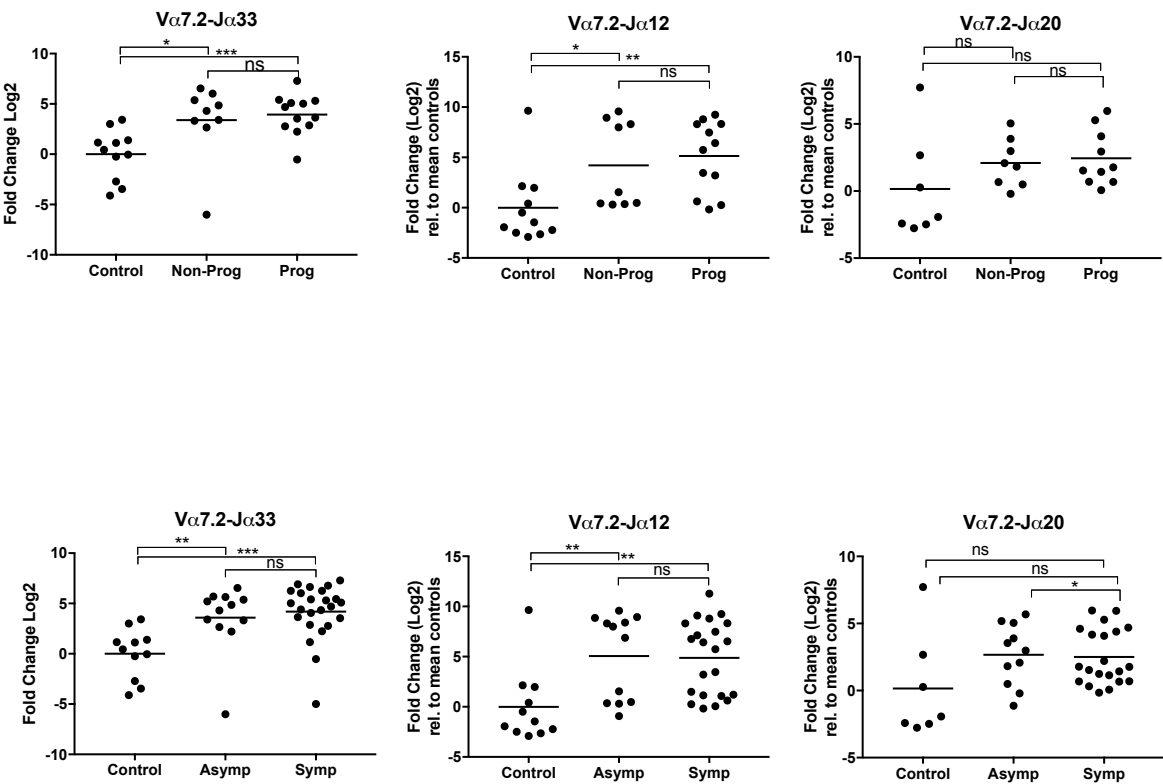

b

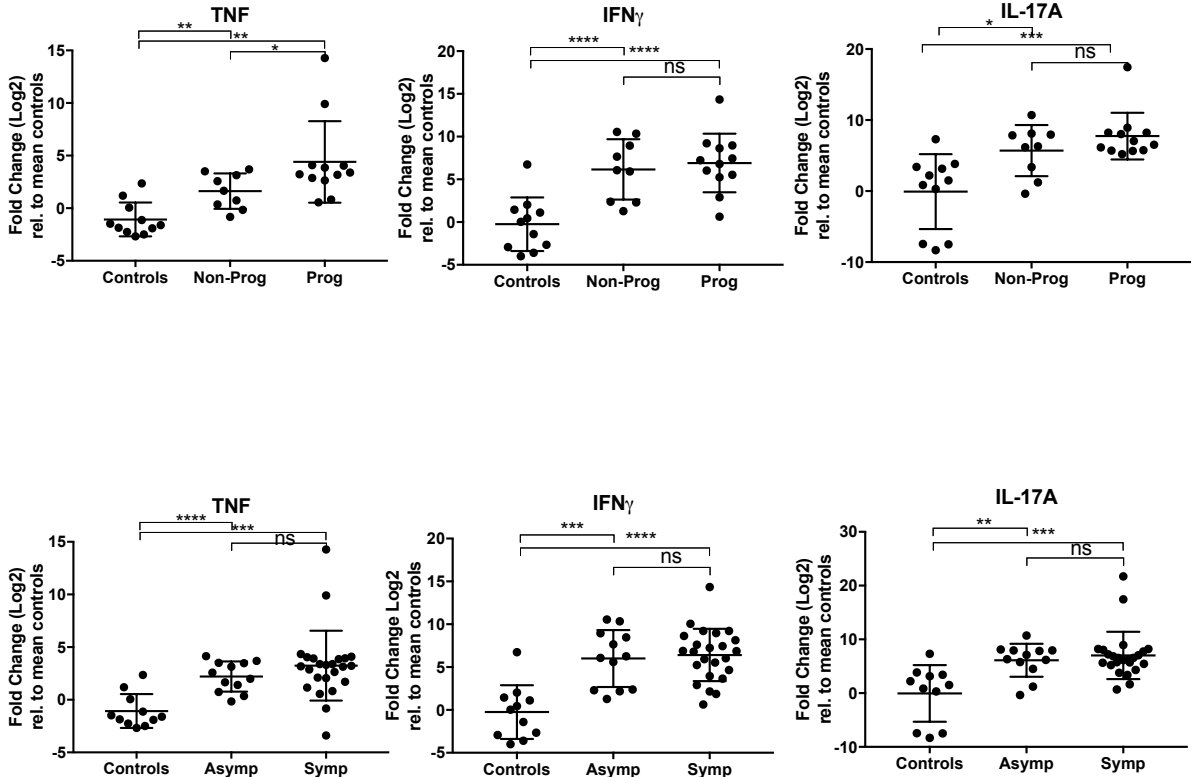

Supplement: Supplementary file 1 — Supplementary Figure I [file 41368_2019_49_MOESM1_ESM.pdf]

Supplementary Figure. II

a

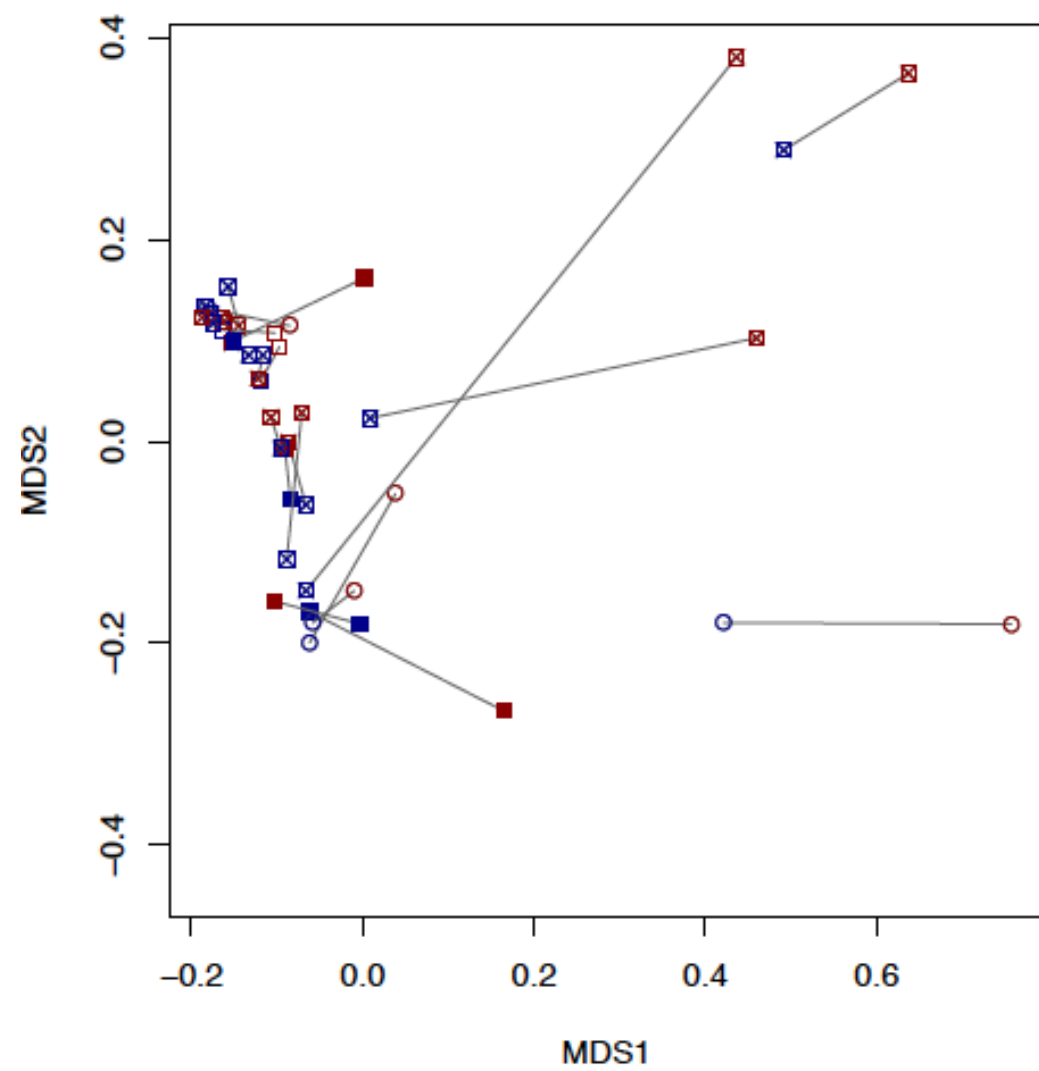

b

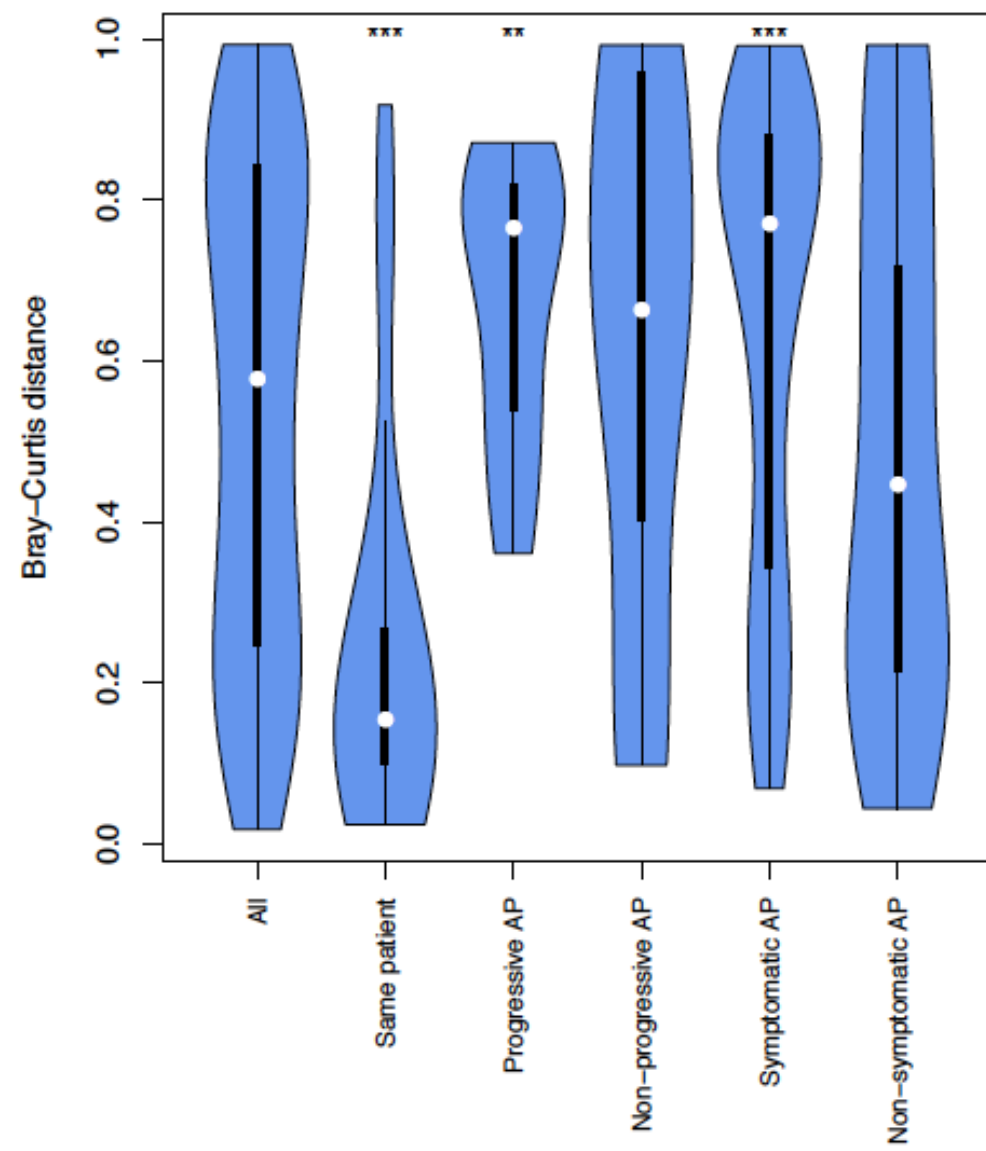

Supplement: Supplementary file 2 — Supplementary Figure II [file 41368_2019_49_MOESM2_ESM.pdf]
