# Supplementary material for: Mucosal-associated invariant T cells and oral microbiome in persistent apical periodontitis
Source: Int J Oral Sci. 2019 May 9;11(2):16. doi: 10.1038/s41368-019-0049-y (PMC6506549; doi:10.1038/s41368-019-0049-y)
Supplement: Supplementary file 3 — Supplementary Figure legend [file 41368_2019_49_MOESM3_ESM.docx]

**Supplementary Figure Ia).** **Comparative analysis of Vα7.2-Jα33, Vα7.2-Jα12 Vα7.2-Jα20,** and **Ib) TNF, IFN-γ and IL-17 expression in AP lesions with or without progression or symptom and gingival control biopsies.** Tissue RNA isolated from non-progressive, or progressive AP lesions, or gingival control tissues (upper panel), and asymptomatic, or symptomatic AP lesions, or gingival tissues (lower panel) were reverse transcribed to cDNA. Targeted qPCR was performed and data are expressed as Log2 fold change relative to GAPDH expression. Statistical analysis was done using Student’s t-tests. ns indicates non-significant, *, **, ***, and **** indicates p-value <0.05, <0.01, <0.001, or <0.0001, respectively.

**Supplementary Figure II. Bray-curtis distance between samples (a)** Non-metric multidimensional scaling of the distance between each sample. Blue symbols: control tissue. Red symbols: AP tissue. Squares: symptomatic cases. Circles: asymptomatic cases. Filled shapes: progressive AP. Empty shapes: non-progressive AP. Cases where the progression status is unknown are marked with an X. Pairs of samples from the same individual are connected by a grey line. **(b)** Violin plot quantifying the distances depicted in (a). ** p<=0.01; *** p<0.001.
